# Digital microfluidic immobilized cytochrome P450 reactors with integrated inkjet-printed microheaters for droplet-based drug metabolism research

**DOI:** 10.1007/s00216-018-1280-7

**Published:** 2018-08-02

**Authors:** Gowtham Sathyanarayanan, Markus Haapala, Iiro Kiiski, Tiina Sikanen

**Affiliations:** Drug Research Program, Division of Pharmaceutical Chemistry and Technology, Faculty of Pharmacy, University of Helsinki, P.O. Box 56, 00014 Helsinki, Finland

**Keywords:** Digital microfluidics, Drug metabolism, Cytochrome P450, Microheater, Microreactor, Enzyme immobilization

## Abstract

We report the development and characterization of digital microfluidic (DMF) immobilized enzyme reactors (IMERs) for studying cytochrome P450 (CYP)-mediated drug metabolism on droplet scale. The on-chip IMERs consist of porous polymer (thiol-ene) monolith plugs prepared in situ by photopolymerization and functionalized with recombinant CYP1A1 isoforms (an important detoxification route for many drugs and other xenobiotics). The DMF devices also incorporate inexpensive, inkjet-printed microheaters for on-demand regio-specific heating of the IMERs to physiological temperature, which is crucial for maintaining the activity of the temperature-sensitive CYP reaction. For on-chip monitoring of the CYP activity, the DMF devices were combined with a commercial well-plate reader, and a custom fluorescence quantification method was developed for detection of the chosen CYP1A1 model activity (ethoxyresorufin-O-deethylation). The reproducibility of the developed assay was examined with the help of ten parallel CYP-IMERs. All CYP-IMERs provided statistically significant difference (in fluorescence response) compared to any of the negative controls (including room-temperature reactions). The average (*n* = 10) turnover rate was 20.3 ± 9.0 fmol resorufin per minute. Via parallelization, the concept of the droplet-based CYP-IMER developed in this study provides a viable approach to rapid and low-cost prediction of the metabolic clearance of new chemical entities in vitro.

**Electronic supplementary material:**

The online version of this article (10.1007/s00216-018-1280-7) contains supplementary material, which is available to authorized users.

## Introduction

Cytochromes P450 (CYPs) are a superfamily of enzymes responsible for the elimination of the majority of drug compounds in humans [1–3]. Understanding the CYP-mediated biotransformation is therefore one of the most crucial steps of drug discovery. Determination of the overall metabolic clearance and identifying both the produced metabolites and the isoenzymes responsible of the elimination should be examined to assess the safety and efficacy of the new drug candidate (i.e., whether it interferes with the elimination of other clinically used drugs or with steroid and fatty acid metabolism) [4]. Although computational methodologies are well established for quantitative structure-activity relationship analyses [4], the selectivity of the enzymatic reactions is challenging to both the computational tools and the non-enzymatic experimental techniques, such as electrochemical oxidation or TiO_2_ photocatalysis [5]. Therefore, rapid, enzymatic in vitro screening methods are of vital importance in drug metabolism research. An important goal in this field is to reduce the consumption of expensive and limited biological resources, such as the hepatic enzyme preparations of human origin, via miniaturization [6–8]. Enzyme immobilization onto a solid support material often improves the enzyme activity and stability and facilitates more straightforward separation (and thus identification) of the unknown reaction products from the incubation matrix [8, 9]. A variety of capillary-based immobilized enzyme reactors (IMERs) have been implemented in the past, including covalent and non-covalent binding to a solid support material and entrapment inside a crosslinked polymer network [10]. Even lateral flow CYP assays [11] and nanoliter-scale microarrays with CYPs encapsulated in as many as 1134 parallel gel compartments patterned on a functionalized glass slide [8] have been reported. Implementation of microfluidic actuation and integration of other sample preparation and separation units with the IMER technology [12] would facilitate adaptation of even more mature lab(oratory)-on-a-chip technology concepts to drug metabolism research.

Digital microfluidics (DMFs) based on electrowetting-on-dielectric is a versatile lab-on-a-chip technology in droplet scale (typically a few hundreds of nL to a few μL droplets) [13]. Automated actuation (e.g., with the open-source DropBot [14]) facilitates rapid dispensing, mixing, and splitting of individual droplets of reagents in parallel, and often in a more straightforward manner than feasible for in-channel microfluidics and sequential operations thereof [15]. Compared with well-plate-compatible technologies, DMF is capable of increasing the levels of assay complexity and customization, e.g., droplet-scale synthesis [16] with CYP screening. DMF devices, typically consisting of a bottom plate (actuation electrodes) and a cover plate (counter electrode), can also be interfaced with all common sensing methods such as optical, electrochemical, and mass spectrometry detection [17]. These features have made DMF a potential tool for performing versatile enzymatic and cell-based assays in a droplet scale in parallel, but with significantly low consumption of (bio)chemicals [13, 18, 19]. Although the feasibility of the DMF technology for a variety of bioanalytical applications is well established [19, 20], the possibilities of performing drug metabolism assays on a DMF platform have not been comprehensively studied. This is likely because of the complexity of the CYP system [21], which comprises of several electron-supplying cofactors (e.g., cytochrome P450 reductase) that contribute to the redox reactions. Also, the facts that all cofactors must be embedded in the lipid environment and kept at physiological temperature have significantly hindered the implementation of microfluidic CYP-IMERs. In this study, we examined the feasibility of DMF for studying drug metabolism in droplet scale by emphasizing the common challenges related to implementation of microfluidic CYP assays. First, miniaturization of CYP reactors down to droplet scale is challenged by the relatively low yield of the CYP reactions, which requires sufficiently high amounts of (immobilized) enzymes to be able to produce detectable amounts of metabolites. To maximize the surface area of droplet-based IMERs, we introduced thiol-rich porous polymer monoliths, cured in situ on the DMF bottom plate, for functionalization with recombinant CYP1A1 (rCYP1A1, an important route for many xenobiotic detoxification and steroid and fatty acid metabolism) via avidin-biotin chemistry. Second, inspired by the printed intelligence [22, 23], we also developed inkjet-printed microheaters for regio-specific heating of the IMERs to physiological temperature, which allowed us to reversibly attach the heaters to the DMF bottom plate and customize the heater capacity and the assay design on demand. Third, we developed a custom protocol for interfacing the DMF-based rCYP assays with well-plate-based fluorescence detection to accommodate with parallel detection of all droplet assays simultaneously. Finally, the method was qualified in terms of detection sensitivity, linearity, repeatability, and specificity using ethoxyresorufin-O-deethylation via rCYP1A1 as the model reaction.

## Experimental

### Materials and reagents

Pentaerythritol tetrakis(3-mercaptopropionate), > 95% (tetra-thiol); 1,3,5-triallyl-1,3,5-triazine-2,4,6(1H,3H,5H)-trione (tri-ene), 98%; β-nicotinamide adenine dinucleotide 2′-phosphate reduced tetrasodium salt hydrate (NADPH); biotin-PEG_4_-alkyne; rhodamine B; magnesium chloride; resorufin; acetone; methanol; ethylene glycol; and chloroform were purchased from Sigma-Aldrich (Steinheim, Germany). Potassium dihydrogen phosphate was purchased from Riedel-de Haën (Seelze, Germany) and dipotassium hydrogen phosphate was purchased from Amresco (Solon, OH). Irgacure® TPO-L (photoinitiator) was donated by BASF (Ludwigshafen, Germany). Streptavidin, Alexa Fluor® 488 conjugate (Alexa-SA), was purchased from Thermo Fisher Scientific (Waltham, MA) and 7-ethoxyresorufin from Toronto research chemicals (Toronto, ON, Canada). The lipids 1,2-dioleoyl-*sn*-glycero-3-phosphoethanolamine, 1,2-dioleoyl-3-trimethylammonium-propane (chloride salt), 1,2-dioleoyl-*sn*-glycero-3-phosphoethanolamine-*N*-(Cap biotinyl) (sodium salt), and 1,2-dioleoyl-*sn*-glycero-3-phosphoethanolamine-*N*-(lissamine rhodamine B sulfonyl) (ammonium salt) were purchased from Avanti Polar Lipids, Inc. (Alabaster, AL). Recombinant cytochrome P450 1A1 (rCYP1A1) (1000 pmol mL^−1^) was purchased from Corning (Wiesbaden, Germany). Water was purified with a Milli-Q water purification system (Merck Millipore, Molsheim, France). All solvents were of analytical or HPLC grade.

SU-8 5 was purchased from Microchem Corp., (Westborough, MA). Cytonix FluoroPel PFC 1604V and PFC 110 fluoro-solvent were purchased from Cytonix LLC (Beltsville, MD). AZ 351B resist developer was purchased from MicroChemicals GmbH (Ulm, Germany).

### Fabrication and operation of the digital microfluidic chips

**Fig. 1:**
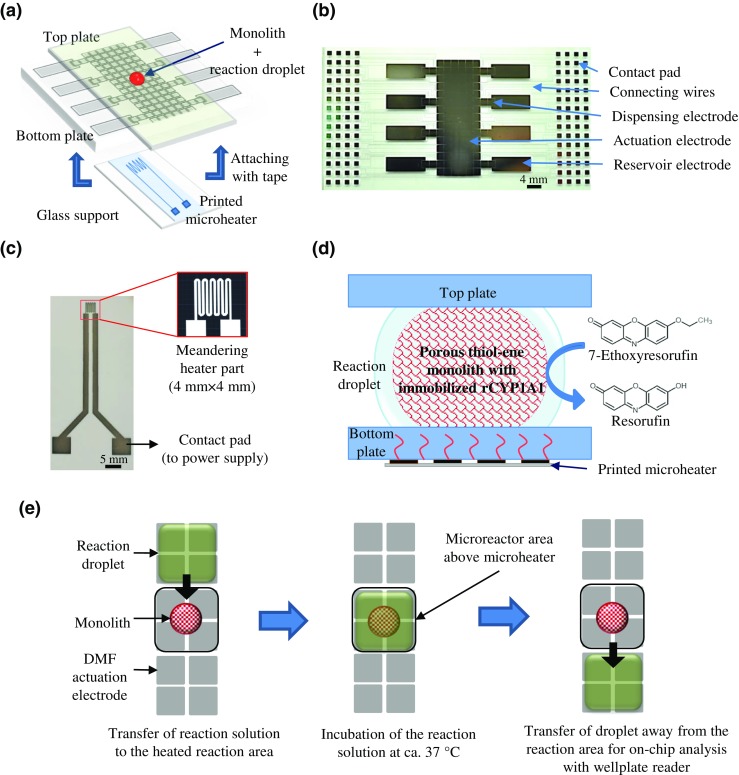
**a** Schematic view of the DMF chip-microheater assembly (not in scale). **b** Photograph of the DMF bottom plate. **c** Photograph of the microheater design used including the contact pads and CAD layout of the meandering heater part (magnified). **d** Schematic illustration of the cross-section of heated CYP-IMER region of the DMF chip. **e** Schematic view of the droplet actuation protocol during the on-chip CYP incubation on the DMF device

The DMF chip design consists of a bottom plate (50 mm × 75 mm) and a cover plate (25 mm × 75 mm), which are assembled on top of each other with double-sided tape (two layers) to leave a 180-μm-thick gap between the plates (Fig. 1a). The bottom plate features an array of 15 × 6 actuation electrodes (each 2 mm × 2 mm) for sample manipulation, eight reservoir electrodes (each 11 mm × 4 mm), and 16 dispensing electrodes (each 2 mm × 2 mm) for dispensing samples from reservoirs to the actuation electrodes (Fig. 1b). In addition, all active electrodes were connected to their individual contact electrodes (1 mm × 1 mm) (via 30-μm-wide connecting wires), which were arrayed on both sides of the bottom plate (Fig. 1b). The bottom plate was fabricated by photolithography and wet etching under non-cleanroom conditions using commercial glass substrates with chromium (100 nm) and AZ 1500 photoresist (530 nm) coatings (Telic Company, Valencia, CA). First, the electrodes were patterned by exposing the AZ resist to UV, through a high-resolution plastic photomask (Micro Lithography Services Ltd., Chelmsford, UK), for 5 s using a Dymax 5000-EC Series UV flood exposure lamp (Dymax Corporation, Torrington, CT) with nominal power of 225 mWcm^−2^. After exposure, the photoresist was developed with AZ 351 B developer (20% in water, *v*/*v*) for 20 s followed by chromium etching in chrome-etchant 3144 Puranal (Honeywell International, Morris Plains, NJ) for ca. 45 s. The resist was released with acetone in an ultrasound bath (5 min). The arrays of contact electrodes on both sides of the substrate were covered with tape and a 8-μm-thick SU-8 layer was spin coated (1600 rpm, 30 s) onto the substrate and cured under UV for 15 s (flood exposure). The SU-8 layer was baked on a hot plate at 65 °C for 2 min and at 95 °C for an additional 2 min before and after the UV exposure. Finally, a 1-μm-thick fluoropolymer layer was spin coated (1% FluoroPel, 1000 rpm, 30 s) onto the SU-8 surface and hard-baked on the hot plate at 150 °C for 30 min. Hydrophilic spots for later integration of the porous polymer monolith plugs were implemented by masking the SU-8 surface during fluoropolymer coating with circular pieces of tape (i.d. 2 mm), which were cut using a biopsy puncher and removed after the hard bake. The cover plate was an indium tin oxide (ITO)-coated glass plate (Structure Probe, Inc., West Chester, PA), which was further coated with the fluoropolymer layer in house like the bottom plate. All DMF operations were performed by applying a driving voltage of 100 to 140 V_rms_ (at 10 kHz) to the electrodes (bottom plate) using a DropBot open-source DMF automation system [14] and grounding the ITO layer (top plate).

### Fabrication and characterization of the microheaters

The microheater design consisted of a meandering, 200-μm-wide line, which covered an area of 4 mm × 4 mm (equal to four actuation electrodes of the DMF chip) and square contact pads at the ends for connecting to the power supply (Fig. 1c). The design was drawn with AutoCAD 2015 (Autodesk Inc., Mill Valley, CA) and printed on a transparent polyethylene terephthalate film (AGIC-CP01A4 Special Coated Paper, AgIC Inc., Tokyo, Japan) using a Brother MFC-J5910DW inkjet printer and AGIC-AN01 Silver Nano Ink (AgIC Inc.). After printing, the heater film was sintered on the hot plate at 100 °C for 2 min. For local heating of the DMF chip, the microheater was assembled on a glass support plate (25 mm × 50 mm) and attached to the bottom plate under the monolith plug (i.e., the CYP-IMER) using double-sided tape.

To characterize the microheater performance, voltage (typically 1.26–2.20 V corresponding to 0.05–0.15 W) was applied to the contact pads and the temperature of the sample droplet was measured by inserting a miniature thermocouple (wire diameter 50 μm, CHAL-002, OMEGA Engineering, Manchester, UK) in the droplet between the DMF top and bottom plates. The temperature was recorded with a Fluke 289 multimeter (Fluke Corp., Everett, WA). In addition, infrared (IR) thermography was used for measuring the temperature distribution on the chip surface. IR thermography was performed with a FLIR A325sc IR camera (FLIR Systems, Inc., Wilsonwille, OR) equipped with a lens with 45° field of view and 10-mm focal length (emissivity of 1.00 was assumed for the glass surfaces). The accuracy of the IR camera with respect to absolute temperature (without calibration) was ± 2 °C. However, since only temperature changes (ΔT) relative to initial room temperature in the beginning of the measurement (as the calibration point) were monitored, the precision (resolution) of the IR-based temperature measurement was solely determined by its noise equivalent differential temperature (NEDT), which was as good as 0.05 °C. The volume loss due to evaporation upon heating was determined by monitoring the size of the fluorescent dyed droplet (0.1 mM rhodamine B in water) under a Zeiss Axioscope A1 upright epifluorescence microscope (Zeiss Finland Oy, Espoo, Finland). A 100-W halogen lamp (HAL 100, Zeiss) was used as the excitation source with bandpass excitation (546 ± 6 nm) and emission (575–640 nm) filters. Fluorescent images were taken every 10 s with a Retiga 4000R digital microscope camera (QImaging, Surrey, BC, Canada) using × 2.5 magnification and were processed with ImageJ software. The volume loss during evaporation was calculated based on the area of the droplet at each time point (the gap between the top and bottom plates was constant).

### Fabrication and functionalization of porous polymer monoliths

The porous polymer monoliths were prepared from a thiol-rich, off-stoichiometric thiol-ene mixture similar to in-channel monoliths in an earlier work [24] by mixing tetra-thiol and tri-ene in a ratio that yielded a 50 mol% excess of thiol functional groups. A total of 5 μL of 10% Irgacure TPO-L in methanol and 2 g of methanol were added to 0.5 g of the thiol-ene mixture. Methanol served as the porogen and, while stirring at 1000 rpm, formed a homogeneous emulsion with the thiol-ene mixture. After stirring (ca. 1 min), 2 μL of the emulsion was pipetted on top of each hydrophilic spot of the DMF bottom plate, after which the plate was immediately covered with a glass plate and cured under UV (through the glass plate) for 1.5 min. After curing, the glass plate was removed and the monoliths were washed thoroughly by flushing with a few milliliters of Milli-Q water.

Next, the monoliths were functionalized sequentially with fluorescent-derivatized (Alexa Fluor) streptavidin and fluorescent-labeled (lissamine rhodamine B) biotinylated recombinant cytochrome P450 (CYP) 1A1 supersomes similar to the previously described protocols by Feidenhans’l et al. (2014) [25] and Kiiski et al. (2016) [26], respectively (Fig. 1d). First, the monoliths were biotinylated by pipetting 2 μL of 1 mM biotin-PEG_4_-alkyne in poly(ethylene glycol) containing 1% (*v*/*v*) Irgacure TPO-L on top of each monolith followed by cross-linking under UV for 1 min. After curing, the monoliths were washed thoroughly with a few milliliters of methanol and then with phosphate-buffered saline (PBS). The excess solvent was allowed to dry after which 2 μL of 0.5 mg mL^−1^ Alexa-Streptavidin (Alexa-SA) in PBS was pipetted on each monolith and incubated at room temperature for 15 min to yield a streptavidin-coated monolith. Small volumes of PBS were added intermittently to avoid complete evaporation of the solvent. After incubation, the monoliths were washed with PBS as described above and functionalized with biotinylated rCYP1A1 supersomes comprising a Lissamine rhodamine B tag. A total of 5 μL of the rCYP1A1 mixture was applied over each monolith and incubated at room temperature for 15 min. Again, small volumes of PBS were added intermittently to prevent drying. After incubation, the monoliths were washed with PBS and care was taken to prevent drying before use to preserve the rCYP1A1 activity. The streptavidin and rCYP1A1 functionalization processes were monitored with fluorescence originating from the Alexa-SA (*λ*_ex_ = 495 nm, *λ*_em_ = 519 nm) and the rhodamine-tagged rCYPs (*λ*_ex_ = 546 nm, *λ*_em_ = 590 nm), respectively.

### Cytochrome P450 assays on chip

The rCYP1A1 activity of the monolith-based IMERs (Fig. 1d) was determined with an ethoxyresorufin-O-deethylase (EROD) assay [27–29]. The EROD assay was performed with 2 μM ethoxyresorufin as the substrate and 0.5 mM NADPH as the co-substrate, both dissolved in 0.1 M potassium phosphate buffer (pH 7.4) containing 5 mM MgCl_2_. A droplet equal to the size of four actuation electrodes (volume ca. 3 μL) of the substrate solution was dispensed on each monolith and incubated at 37 °C for 30 min (Fig. 1e). The incubation was initiated by applying a voltage corresponding to a heating power of 0.1 W to the printed microheater and terminated by bringing the sample droplet away from the monolith and the heated region. To compensate for volume loss due to evaporation, droplets equal to the size of one (ca. 0.8 μL) or two (ca. 1.6 μL) actuation electrodes of potassium phosphate buffer were added to the reaction solution during incubation at room temperature and under heating (37 °C), respectively. The volume loss due to evaporation was determined as described in section “Evaporation effects” and was shown to be linear over time, which facilitated precise calculation of the required compensation volume. After incubation, the CYP1A1 activity was determined by quantifying the resorufin concentration by fluorescence (*λ*_ex_ = 570 nm, *λ*_em_ = 590 nm) on the well-plate reader. The specificity of the method was examined with the help of four different kinds of negative control incubations, by omitting the substrate, the co-substrate, or the immobilized enzyme, or by carrying out the enzyme incubation at room temperature.

### Fluorescence measurements

The fluorescence originating from Alexa-SA (*λ*_ex_ = 495 nm, *λ*_em_ = 519 nm) and the lissamine rhodamine B-labeled rCYPs (λ_ex_ = 546 nm, *λ*_em_ = 590 nm) was monitored by using the Zeiss AxioScope A1 upright epifluorescence microscope with HAL100 halogen lamp as the fluorescence excitation source (Zeiss Finland Oy, Espoo, Finland). The enzymatic reaction product, resorufin (*λ*_ex_ = 570 nm, *λ*_em_ = 590 nm), was quantitated by using the Varioskan LUX well-plate reader (Thermo Fisher Scientific, Vantaa, Finland). For this purpose, the DMF chips were fixed on top of the 96-well-plate lid as described in the Electronic Supplementary Material (ESM) Fig. S1 and multipoint fluorescence analysis was performed through the transparent DMF cover plate. The method was qualified according to the ICH guidelines [30] by determining the lower limits of resorufin detection (DL) and quantitation (QL) based on the standard deviation of the response and the slope of a specific calibration curve in the range of DL. The linearity was established between 0.15 and 15 pmol resorufin and the precision (repeatability) of the assay was determined from three replicates at three different concentration levels (0.15, 1.5, and 15 pmol). All resorufin standards were dissolved in the incubation matrix containing 0.1 M potassium buffer (pH 7.4) containing 5 mM MgCl_2_, 2 μM ethoxyresorufin (substrate), and 0.5 mM NADPH (co-substrate).

## Results and discussion

### Characterization of the inkjet-printed microheaters

In this work, we developed a concept for interfacing inkjet-printed microheaters with any common DMF chip design to facilitate rapid, regio-specific heating of the DMF chip on demand. CYPs are particularly sensitive to temperature changes and maintain their maximum activity only in a very narrow temperature range [31]. Performing CYP assays on a DMF chip thus requires rapid and precise heating of the reaction solution to ca. 37 °C. Although biological temperatures can be reached by placing the entire DMF chip inside an incubator [18], integration of regio-specific microheaters provides more efficient heat transfer, and thus more precise control over heating time, which is crucial in enzyme kinetic determinations. The possibility for local heating also allows greater flexibility in terms of using heat-inactivated reagents, such as the co-substrate NADPH [32] required in CYP assays.

**Fig. 2:**
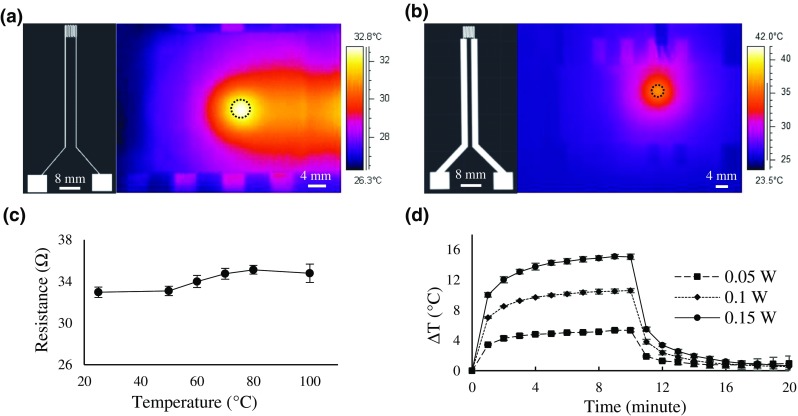
**a**, **b** Infrared (IR) thermographs illustrating the temperature distributions on the DMF chip surface when ca. 3 μL water droplet (circled, area ca. 4 × 4 mm^2^) was heated with the corresponding heater design featuring either 200-μm-wide (**a**) or 2-mm-wide (**b**) connecting wire. **c** Effect of temperature on the resistance of inkjet-printed microheaters. **d** Thermal stabilization curves for the heating and cooling cycles obtained at different heating powers for a sample droplet equal to size of four actuation electrodes (volume ca. 3 μL). The error bars represent the deviation between *n* = 5 parallel microheaters (**c**) and *n* = 3 repeated experiments (**d**)

To address the limited durability, often associated with inkjet-printed metal patterns, and to evaluate whether the inkjet-printed heaters meet the robustness required in biological experiments (at 37 °C), we characterized the performance of the microheaters by IR thermography and resistivity measurements. Although IR imaging provides a fast, high-resolution approach for measuring temperature differences on surfaces [33], it has been minimally used for temperature mapping of microfluidic devices. Here, IR imaging provided a convenient approach to design optimization on the basis of heat distribution plots (Fig. 2). For example, to prevent resistive heating elsewhere but the reaction zone, the resistivity of the connecting wires will need to be reduced to minimum, as illustrated in Fig. 2a, b. Thus, in our design, we used 2-mm-wide connecting wires, while the heated part featured a meandering, 200-μm-wide line corresponding to the area of ca. four DMF actuation electrodes (4 mm × 4 mm). As illustrated in Fig. 2b, the temperature reaches ca. 36 °C only locally on top of the printed microheater, while other areas remain close to room temperature. This significantly increases the efficiency of heating and also eliminates the risk of premature degradation of the co-substrate, NADPH.

Next, to characterize the durability of the inkjet-printed microheaters upon heating, the effect of sintering on the resistance was determined by heating the microheaters (*n* = 5) on a hot plate by gradually increasing the temperature at 5-min intervals (Fig. 2c). Both the printed silver ink and the support plastic sheet material remained undamaged and intact upon heating up to 100 °C. The resistance of the microheater slightly increased with increasing temperature. As expected, after sintering (cooling back to room temperature), the average resistance was slightly decreased (30.8 ± 0.4 Ω, *n* = 5) compared to that before sintering (33.0 ± 0.5 Ω, *n* = 5) due to compacting.

Finally, to determine the heating and cooling times required to reach plateau level at constant applied heating power, the temperature difference (ΔT) versus time was measured at constant heating power of 0.05, 0.1, and 0.15 W (Fig. 2d). At these applied power values, temperature differences of about 6, 10, and 15 °C were reached, respectively. In all cases, the temperature increased rapidly within the first couple of minutes and reached a plateau level in ca. 10 min (*n* = 3 heaters). While the heating power mainly determined the plateau level (ΔT), the characteristic stabilization time was found to be independent of the heating power (Fig. 2d). For the setup and conditions (RT) used in this study, a heating power of 0.1 W was required to reach physiological temperatures (ca. 37 °C) inside the droplet. However, the rapid prototyping by inkjet printing facilitates flexible redesigning of the heater geometry for larger and smaller thermal masses (i.e., different-sized sample droplets), while meeting the required robustness in the range of physiologically relevant temperatures. Compared with previously reported approaches making use of Peltier element [34] or fully integrated, lithographically defined microheaters [35, 36], our setup benefits from simplicity in terms of fabrication (rapid non-cleanroom method) and customization of the assay design (incl. the heated area and the heating power) by simply redesigning and printing new geometry on demand.

### Evaporation effects

**Fig. 3:**
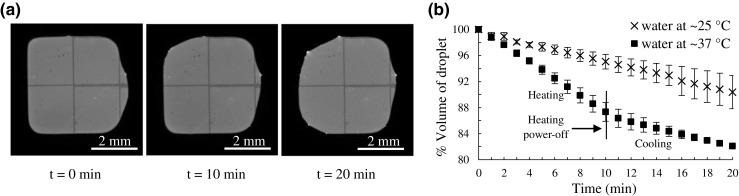
**a** Fluorescent images illustrating the volume loss of a sample droplet due to evaporation upon heating to 37 °C at *t* = 0, 10, and 20 min. **b** Effect of heating (to 37 °C) and cooling back to room temperature on the volume loss of the water droplet (ca. 3 μL) (■) in comparison to the constant decrease in volume at RT (×)

Although the cover plate generally protects the DMF chips from significant evaporation at room temperature, the sample (volume) loss upon heating to 37 °C was visible in about 10–20 min even by eye (Fig. 3a). Therefore, we determined the percentage volume loss over time from fluorographs as illustrated in Fig. 3a. The sample volume (containing fluorescent dye) was calculated at each time point at room temperature (ca. 25 °C) and upon heating (0.1 W, ca. 37 °C) on the basis of the droplet area, the droplet height being constantly ca. 180 μm. At room temperature, the volume loss was about 5% within 10 min, whereas about 12.5% volume loss was observed during the same period of time when a heating power of 0.1 W was applied (Fig. 3b). During the cooling phase (from 37 °C to room temperature), the evaporation followed a similar linear trend as that at room temperature (i.e., 5% within 10 min). Conveniently, with constant applied power, the volume loss was linear with time, which facilitated precise calculation of the needed compensation volume for the later enzyme reactions. By DMF actuation, an appropriate buffer volume could be precisely dispensed to the reaction solution during the reaction and before fluorescence detection. In this manner, the fluctuation in the substrate and co-substrate concentrations during the reaction could be reduced to the minimum in order to keep the reaction conditions somewhat constant over time.

### Porous thiol-ene polymers as solid supports to enzyme immobilization

**Fig. 4:**
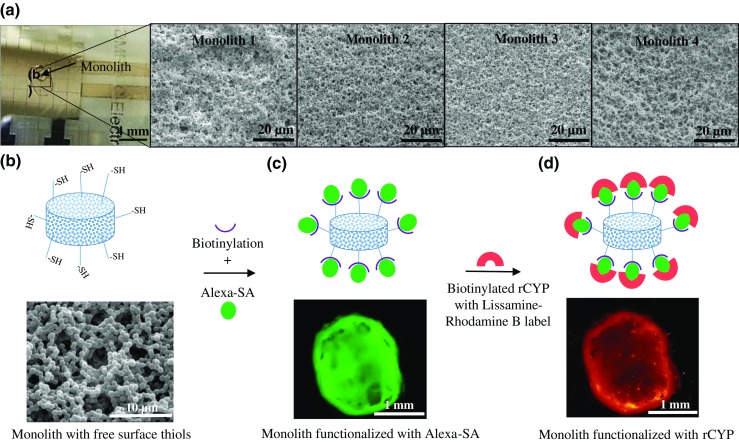
(a) Photograph of monolith on the DMF chip and scanning electron microscope (SEM) images of the fine structure of the porous thiol-ene network of the monolith plugs (*n* = 4 monoliths). Schematic illustration of the CYP immobilization process with (b) SEM image of a thiol-ene monolith and fluorescent images of the monolith functionalized with (c) Alexa-SA and (d) rCYP

The yield (maximum rate) of the CYP assays is generally slow which requires sufficiently high amount of enzymes in order to produce detectable amounts of metabolites in small reaction volumes. In this study, we explored the possibility to use in situ fabricated porous, thiol-ene-based monoliths as solid supports for CYP immobilization via biotin-avidin chemistry. Thiol-ene “click” chemistry conveniently permits UV curing at relatively high wavelengths (upon addition of a photoinitiator) through a standard microscope glass and the use of methanol as the porogen [24], which obviates the need for harsh and toxic chemicals. As a result, uniform porosity with a very large surface-to-volume ratio was obtained (Fig. 4(a)) with uniformly packed beads (diameter ca. 0.5–1.0 μm), similar to those previously reported for thiol-ene-based in-channel monoliths [24]. Compared with microchannel packing, implementation of porous thiol-ene monoliths on an open-to-air DMF bottom plate was significantly more straightforward and did not suffer from, for example, clogging problems typically associated with in-channel monoliths unless the fabrication process is carefully optimized. The microscale porosity between parallel monolith plugs was also shown to be relatively uniform as illustrated in Fig. 4(a). With the bottom plate design used in this study, up to four to five parallel monolith plugs (reactors) could be prepared on a single DMF chip, though less parallel reactors allowed easier sample dispensing protocols. Typically, three parallel reactors per chip were implemented for CYP assays in this study.

As a result of the off-stoichiometric monomer ratio, the porous thiol-ene monolith bears free thiols on the surface after curing [22] which allowed us to functionalize the monolith sequentially with alkyne-biotin and Alexa-SA, and eventually with rhodamine-tagged, biotinylated rCYP1A1. To ensure the success and quality of the functionalization process, the fluorescent intensities of Alexa-SA (Fig. 4(c)) and rCYP1A1s (Fig. 4(d)) were qualitatively monitored by fluorescence microscopy. Unless used immediately, the chips could be stored overnight in the fridge inside a petri dish with dampened wipes (humidity chamber), while the monoliths were covered with PBS dispensed between the top and bottom plates until further experiments. Alternatively, to allow dry storing, the functionalization of the monolith can be ceased after biotinylation and continued from Alexa-SA on the day of use.

Due to the opaqueness of the porous monolith, quantitation of the immobilized rCYP1A1 based on the rhodamine tag was not feasible, but had to be performed indirectly via determination of the rCYP1A1 model EROD activity (ethoxyresorufin-O-deethylation), as described in the next section “Determination of the on-chip cytochrome P450 activity.” On the basis of the EROD activity, the average amount of immobilized P450 was ca. 0.88 ± 0.39 fmol (*n* = 10 CYP-IMERs), assuming that the enzyme activity of the immobilized rCYP1A1 is similar to that of the soluble rCYP1A1 (23 pmol/min/pmol P450, Corning). The relatively large variation (ca. 44%) in the amount of immobilized rCYP1A1 between parallel (*n* = 10) CYP-IMERs is likely resulting from the fact that the total surface area of the porous monolith plug could not be precisely controlled (a common drawback of all monolith systems). Therefore, the developed assay is not readily feasible for parallel quantitative analyses, such as enzyme kinetic determinations or inhibition (e.g., IC50) screening. Instead, the developed assay fits very well with droplet-based synthesis of new chemical entities, such as the work by Jebrail et al. [16], by providing a platform for immediate analysis of the main metabolism routes (responsible isoenzymes) in compatible, small volumes.

### Determination of the on-chip cytochrome P450 activity

**Fig. 5:**
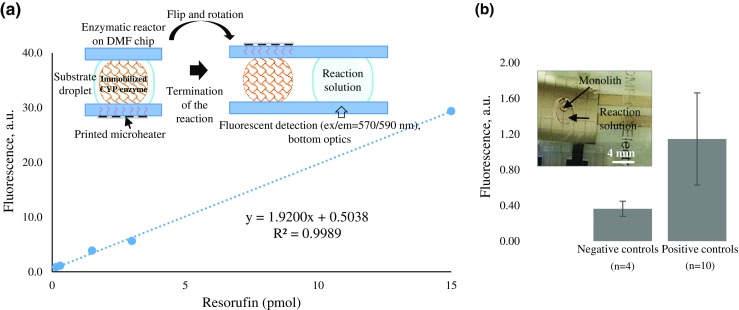
**a** Resorufin calibration curve and schematics of the fluorescence quantification by well-plate reader. **b** The fluorescence signal (*λ*_ex_ = 570 nm, *λ*_em_ = 590 nm) of the reaction solutions (positive controls, *n* = 10, and negative controls, *n* = 4 CYP-IMERs) extracted from the monolith-based rCYP1A1-IMERs and analyzed on chip with the well-plate reader

The CYP1A1 activities of the monolith-based IMERs were determined with an EROD assay (Fig. 5a) using 2 μM ethoxyresorufin as the substrate and 0.5 mM NADPH as the co-substrate, which yielded 0.61 ± 0.27 pmol (*n* = 10 reactions) resorufin as the metabolite. Despite the relatively small size and volume of the droplets, the multipoint fluorescent measurement protocol developed for the commercial well-plate reader (ESM Fig. S1) allowed us to perform rapid analysis of resorufin in the parallel reaction solutions with sufficiently good sensitivity (DL = 36 fmol and QL = 109 fmol). The linearity (*y* = 1.92*x* + 0.50, *R*^2^ = 0.9989, range 0.15–15 pmol resorufin) of the method was also good with high precision over the entire range (Fig. 5a). The repeatabilities of 0.15, 1.5, and 15 pmol resorufin (in the incubation matrix) were 2.6, 3.0, and 4.8% RSD (*n* = 3 measurements each), respectively. The background interference arising from dummy areas (around the sample droplet) could be completely avoided by defining the measurement area with the help of the well-plate reader software. The CYP reaction time, on the other hand, could be precisely determined by dispensing the substrate solution into the monolith (initiation) and bringing it away from the monolith and the heated region (termination). Finally, the specificity of the method was assessed by performing the EROD reaction in ten parallel CYP-IMERs and comparing their resorufin responses (*λ*_ex_ = 570 nm, *λ*_em_ = 590 nm) with those of the negative control incubations carried out without the substrate, the co-substrate, the immobilized enzyme, or heating (room temperature reaction). As a result, the specificity of the method was shown to be good with statistically significant (*p* = 0.001) difference between the average fluorescence response of the CYP assays (*n* = 10) and the negative control incubations (*n* = 4) (Fig. 5b). The metabolite production rate was calculated on the basis of the resorufin concentration in the sample droplet and was on average 20.3 ± 9.0 fmol min^−1^ (*n* = 10 CYP-IMERs). Via redesigning and arraying (multiple CYP-IMERs on a single chip), the developed protocol is most feasible for rapid, qualitative in vitro identification of the responsible CYP isoenzymes that contribute to a drug candidate’s elimination in vivo. Namely, with the aim of identifying the metabolic pathways of a new chemical entity, each of the individual IMERs can also be functionalized with a different, clinically relevant CYP isoform so that all main isoenzyme(s) can be simultaneously screened in order to identify the ones primarily responsible for the compound’s elimination in the body prior to more detailed characterization of the compound’s metabolic profile. However, implementation of quantitative enzyme kinetic or inhibition assays would require further optimization of the enzyme immobilization protocol toward better repeatability, whereas identification of the produced metabolites would necessitate coupling of the DMF assay to a mass spectrometer, as demonstrated in [37, 38].

## Conclusions

We implemented droplet-based CYP-IMERs on DMF devices to permit rapid, on-chip metabolic profiling of drugs and other alike xenobiotics in vitro. All critical aspects related to CYP assays (including the narrow temperature range and slow kinetics) were carefully addressed in the assay design. To address the low yield (slow kinetics) of the CYP reactions, thiol-ene-based porous polymer monoliths were implemented on DMF assays to facilitate significant increase in surface area for CYP immobilization via biotin-avidin chemistry. Here, ca. 0.88 fmol rCYP1A1 was immobilized per IMER producing ca. 0.61 pmol resorufin per reaction, on the average. For regio-specific control of the reaction temperature, we used inkjet-printed microheaters, which allowed customization of the heater capacity and the assay geometry on demand, while maintaining performance similar to integrated, lithographically patterned microheaters. Compared with heated incubator (used in cell assays, for example), the inkjet-printed microheaters provide much shorter thermal stabilization time, which is crucial in order to control the enzymatic reaction time (typically minutes to tens of minutes) with sufficient precision. In addition to significantly reduced reagent consumption (e.g., over conventional well-plate assays), the DMF-based assay benefits from automated handling of reagents and reaction solutions which facilitates arraying of multiple parallel reactions. Most importantly, however, DMF technology enables high level of systems integration and customization of the CYP assays, including possibilities for on-chip synthesis of new chemical entities [16] prior to their CYP interaction screening and for online separation [39] and identification of unknown metabolites by mass spectrometry [37]. Furthermore, all subunits of the developed concept, such as the DMF device (for sample manipulation), the porous polymer monoliths (for enzyme immobilization), and the microheaters (for locally maintaining physiological temperature), could be fabricated and integrated in non-cleanroom conditions and the enzymatic products analyzed with a commercial well-plate reader, which not only significantly reduces the cost but also makes the technology feasible for use in common laboratory conditions.

## Electronic supplementary material

ESM 1 (PDF 148 kb)
