# Supplementary material for: Digital microfluidic immobilized cytochrome P450 reactors with integrated inkjet-printed microheaters for droplet-based drug metabolism research
Source: Anal Bioanal Chem. 2018 Aug 2;410(25):6677–87. doi: 10.1007/s00216-018-1280-7 (PMC6132693; doi:10.1007/s00216-018-1280-7)
Supplement: Supplementary file 1 — (PDF 148 kb) [file 216_2018_1280_MOESM1_ESM.pdf]

Analytical and Bioanalytical Chemistry

Electronic Supplementary Material

Digital microfluidic immobilized cytochrome P450 reactors with integrated inkjet-printed microheaters for droplet-based drug metabolism research

Gowtham Sathyanarayanan, Markus Haapala, Iiro Kiiski, Tiina Sikanen

Assembling digital microfluidic devices in a commercial well-plate reader

For multipoint fluorescence analysis, the DMF chips were fixed on top of the 96-well plate lid (transparent) using tape. Sample droplets were aligned along the wells with the aid of markings on the lid. The lid was then inverted and rotated 180° and placed on top of the support tray inside the wellplate reader (Fig. S1). Multipoint fluorescence analysis was performed using the bottom optics through the DMF top plate. Fluorescence intensity was recorded at nine points (3×3 matrix) along the well/droplet and then averaged.

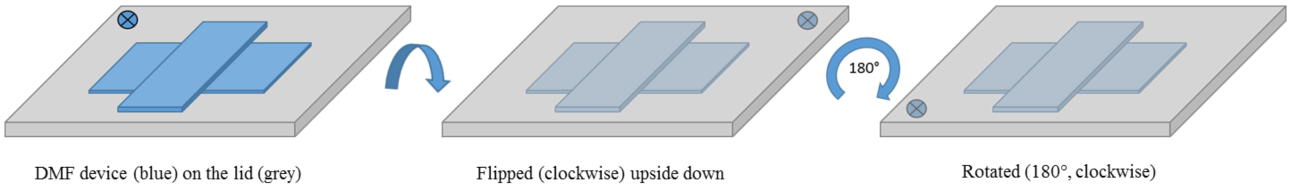

Fig. S1 Schematic representation of flip and rotation of the DMF device taped on a 96-wellplate lid before placing inside the plate reader tray.
